# A cross-sectional study describing factors associated with utilisation of GP services by a cohort of people who inject drugs

**DOI:** 10.1186/1472-6963-14-308

**Published:** 2014-07-16

**Authors:** Dhanya Nambiar, Mark Stoové, Paul Dietze

**Affiliations:** 1 Centre for Population Health, Burnet Institute, 85 Commercial Rd, 3004 Melbourne, VIC, Australia; 2 Department of Epidemiology & Preventive Medicine, Monash University, GPO Box 2284, 3001 Melbourne, Victoria, Australia

**Keywords:** Injecting, General practice, Healthcare, Utilisation, Primary care

## Abstract

**Background:**

People who inject drugs (PWID) use healthcare services, including primary care, at a disproportionately high rate. We investigated key correlates of general practitioner (GP) related service utilisation within a cohort of PWID.

**Methods:**

Using baseline data from a cohort of 645 community-recruited PWID based in Melbourne, Victoria, we conducted a secondary analysis of associations between past month use of GP services unrelated to opioid substitution therapy (OST) and socio-demographic and drug use characteristics and self-reported health using multivariate logistic regression.

**Results:**

Just under one-third (29%) of PWID had accessed GP services in the month prior to being surveyed. Participants who reported living with children (adjusted odds ratio, AOR 1.97, 95% CI 1.04 - 3.73) or having had contact with a social worker in the past month (AOR 1.92, 95% CI 1.24 - 2.98) were more likely to have seen a GP in the past month. Participants who were injecting daily or more frequently (AOR 0.50, 95% CI 0.30 - 0.83) or had a weekly income of less than $400 (AOR 0.59, 95% CI 0.38 - 0.91) were less likely to report having seen a GP in the past month.

**Conclusions:**

Our sample frequently attended GP services for health needs unrelated to OST. Findings highlight both the characteristics of PWID accessing GP services and also those potentially missing out on primary care and preventive services.

## Background

People who inject drugs (PWID) are at elevated risk of acute and chronic health conditions including dermatologic disease, pulmonary and respiratory complications, psychiatric illness, gastrointestinal illness, genitourinary disease, blood-borne viruses (BBV), circulatory complications, asthma, diabetes mellitus and hypertension [1-4]. These conditions can be exacerbated further by the social conditions commonly experienced by PWID such as homelessness, unemployment, lack of financial and social support, violence and stigma and discrimination [5]. Personal characteristics such as age also relate to risk, with young and new initiates to injecting more likely to borrow and lend used injecting equipment and practice unsafe sex, and less likely to test for BBVs compared to older, more experienced PWID [6]. While these risk exposures and health conditions indicate elevated need for health services, the patterns and types of health services used by PWID remain unclear in the Australian context. A recent Australian study reported general practitioners (GPs) the most commonly accessed health service in the past year (64%), followed by PWID-specific primary health care services and emergency services [7]. While rates of GP access in this study were comparable to use in the general Australian population, almost half the study population was on opioid substitution therapy (OST) and the average age of participants was 37 years old. Previous research indicates that older PWID are more likely to engage in health services [8,9]. There has been limited opportunity to study younger PWID in Australia within this context.

The social, health and political contexts of injecting drug use means access to health services by PWID can be appropriately positioned within the Behavioural Model of Andersen and Newman [10]. This well-validated model shows health service utilisation as underpinned by barriers and enablers, and mediated by individuals’ need for care [11]. Although an individual’s perceived need for health care drives motivation to use health services, other factors influence actual utilisation. These predisposing characteristics may include demographic features, health beliefs, social structure, substance use, personal, family and community resources, and factors related to perceived need. Among PWID, factors such as prioritising social needs above health needs, self-perceived health, reluctance to disclose drug use and distrust of service providers, social isolation, transport, service delivery models, and punitive measures around child welfare [2,12-14] have been identified as factors that might ‘predispose’ GP service utilisation. These barriers may underpin the delays in accessing primary health services [2] and related frequent use of emergency services [15], as well as low continuity of care [16].

In Victoria, the majority of primary care delivered to PWID is facilitated through GP services [17]. To address access barriers associated with traditional GP services and provide or facilitate referral to targeted services to meet the specific needs of PWID, primary health care services that cater specifically to PWID were introduced in Victoria in 1999 [18,19]. Located in traditional street drug market "hotspots" in Melbourne, Victoria has five such state-funded free clinics providing primary health care and harm reduction services (including Needle/Syringe Exchange Programs (NSP) and, at some locations, Opioid Substitution Therapy (OST) prescribed by GPs). The objective of these services was to enable a coordinated and comprehensive primary health care response to enhance the health and welfare of PWID who were reluctant to utilise mainstream services [19]. The primary health care setting provides a contact opportunity and service system entry point with this otherwise hard-to-reach population where they can be identified and engaged in harm reduction [20-22]. Distinguishing between dependence-related GP services (primarily opioid substitution therapy) and general health services serves to understand if PWID who are not accessing OST have sufficient access to preventive health services provided by GPs.

Drawing from broader literature around health service utilisation among PWID, our study was designed to examine factors associated with access to general health services through GP service utilisation among a cohort of young PWID in Melbourne with a view to understanding the barriers and enablers to service access.

## Methods

A sample of PWID was recruited between November 2008 and March 2010 in Melbourne, Australia as part of the Melbourne Injecting Drug User cohort study (MIX) that has been described in detail elsewhere [23]. Briefly, participants were individuals aged between 18 and 30 years old (these age criteria included to focus on newer, younger initiates into injecting drug use than those typically recruited in studies of Australian PWID e.g. [24]), who reported injecting either heroin or methamphetamines at least monthly over the previous six months and were able to provide a valid Medicare card number. The purpose of the study was to examine trajectories of injecting drug use among PWID. Participants were recruited using a combination of Respondent Driven Sampling (RDS), street outreach and snowball sampling to ensure maximum participation and representativeness. Participants completed a researcher-administered questionnaire that covered socio-demographics, drug use and drug market access, health and social functioning and health service utilisation. Validated instruments included the AUDIT-C [25], the Personal Wellbeing Index [26,27] as well as the SF8 Health Survey [28]. Participants were reimbursed AUD$30 in cash for their time and out-of-pocket expenses in completing the questionnaire. The study was approved by the Human Research Ethics Committee of the Victorian Department of Health and Monash University Human Research Ethics Committee.

### Measures

Based on the concept of utilisation as one component of healthcare access [29,30], we measured exposures at the individual level associated with utilisation of GP-related services. GP-related service utilisation was defined as accessing a GP in the past month at either general or PWID-specific health centres (Table 1). The outcome variable was derived from asking participants if they had accessed general GP and/or PWID-specific primary health care services in the past month. This was followed by a description of what services were used, such as drug dependence or other services. PWID-specific primary health care services were located in settings that also provided NSP, counselling, allied health, community, welfare and justice services as well as referrals. Participants who indicated they accessed these services were asked about what services they received during their visit, allowing the identification of service contacts at PWID-specific primary care services that were GP-related. Relevant GP-related descriptions included attendance for wound care and injuries, BBVs, pregnancy, general health visits and health referrals. General GP settings may also provide drug dependence services such as OST. OST visits that were concurrent with primary health care visits at either general or PWID-specific primary health care services were included in the analysis but services accessed exclusively for drug dependence (including OST) were not included.

**Table 1:** Health service utilization in the past month by setting

**Outcome**	**General GP settings**	**PWID-specific PHC settings**	**Both**	**None**
All service access	306 (47.4)	33 (5.1)	63 (9.8)	243 (37.7)
GP-related services	155 (24.0)	20 (3.1)	14 (2.2)	456 (70.7)

Exposure variables explored as correlates of GP service utilisation, described in Table 1, were selected based on previous literature on health-related outcomes within the study population [31,32] and those related to a revision of the Behavioral Model of Andersen and Newman for Vulnerable Populations [33]. Low income was defined as a weekly income below $400; the mean income of low income households [34].

### Analysis

Using logistic regression, we explored the bivariate association between past month use of GP services and a range of exposure variables. Associations significant at the p < 0.1 level in bivariate analysis were then included in a multivariate logistic regression model to identify independent correlates of GP service uptake (p < 0.05). Multicollinearity among variables included in the final model was examined and all Variance Inflation Factors (VIF) were within acceptable limits. Model fit was examined using Pearson chi-square goodness-of-fit test. Age, gender and concurrent OST were included in all final models on an *a priori* basis. Analysis was conducted using Stata 11.1 (Statacorp LP, Texas, USA).

## Results

### Sample characteristics

Of the 688 people in the study, 43 did not have complete data on GP service utilisation in the past month and were excluded from the analysis. The remaining 645 respondents were largely male (67%) and born in Australia (81%). Over half of the sample was aged between 25 and 29 years with a median of 27 years. Unstable accommodation (e.g., boarding houses, squatting, homelessness) was reported by 19% of the sample and 34% had less than Year 10 education. The majority of participants (81%) fell within the Australian Bureau of Statistics [35] definition of low income. Over 80% of respondents reported heroin injection in the past month and 53% reported arrest in the past 12 months. More than half of participants (59%) were recruited through snowball sampling, with an additional 38% through RDS and the remaining 3% through outreach.

Of those reporting a testing history (92% for hepatitis C and 88% for HIV) and knowing their results, 52% reported a positive hepatitis C result and 1% reported a positive HIV result. Approximately 44% of participants reported at least one general health problem such as asthma, a current sexually transmitted infection, sleeping difficulties, or mental health problems. The mean self-perceived wellbeing of the sample as measured by the Personal Wellbeing Index was 54.3 (SD 19.0), some twenty points lower than the Australian population mean of 75.4 (SD 12.8) [36].

### Service utilisation

There were 402 (62%) participants who accessed either general GP or PWID-specific primary health care settings in the past month (Table 1). These services include both OST and GP-related services. Among the 645 participants reporting on GP utilisation, 29% had at least one visit to a GP for a health-related reason in the past month that was unrelated to OST, defined as a GP-related service. Most (82%) GP-related visits occurred in general clinic settings. The majority (69%) of respondents who had not visited a GP in the past month did not access hospital, emergency, ambulance, dental, specialist or other services in the past month either.

### Factors associated with GP service utilisation

Table 2 summarises the results of the regression analyses for variables included in this study. Socio-demographic characteristics were only significantly associated with GP service utilisation at the bivariate level, where participants who were female, employed and living with children were more likely to report GP access. In contrast those born in Vietnam (compared to Australia) and from non-English speaking backgrounds were less likely to report GP access in the past month. Of note was the relationship between use of other health services and access to GP services. Although the association was not significant in the final model, the bivariate trends indicated that respondents who used multiple health services were likely to have used GP services as well.

**Table 2:** **Correlates of GP service use in the past month**^**a**^

**Variable**	**n (%)**	**Unadjusted OR**	**Adjusted OR**^**b**^
		**(95% CI)**	**(95% CI)**
**Female**	210 (32.6)	1.84 (1.29 – 2.62)	1.30 (0.86 – 1.98)
**Employed**	85 (13.2)	1.86 (1.16 – 2.97)	1.44 (0.82 – 2.55)
**Low income group**	526 (81.5)	0.50 (0.33 – 0.75)	0.50 (0.30 – 0.83)
**Current stable accommodation**	514 (79.7)	0.96 (0.62 – 1.46)	-
**Education**			
<Year 10	219 (33.9)	1.29 (0.88 – 1.89)	-
Year 10-11 (ref)	292 (45.3)	1	-
Year 12 or higher	134 (20.8)	1.21 (0.77 – 1.90)	-
**Failure at school**	193 (29.9)	0.99 (0.69 – 1.44)	-
**Living with children**	57 (8.8)	2.37 (1.36 – 4.10)	1.97 (1.04 – 3.73)
**Country of birth**			
Australia (ref)	522 (80.9)	1	1
Vietnam	46 (7.13)	0.33 (0.14 – 0.79)	0.72 (0.24 – 2.14)
Other	77 (11.9)	0.77 (0.45 – 1.33)	0.98 (0.53 – 1.82)
**Non-English speaking background**	94 (14.6)	0.45 (0.25 – 0.79)	0.79 (0.38 – 1.62)
**Identify as Aboriginal or Torres Islander**	38 (5.9)	0.98 (0.47 – 2.02)	-
**Age at interview**			
<20	34 (5.3)	2.0 (0.98 – 4.12)	1.75 (0.80 – 3.84)
20-24	153 (23.7)	1.1 (0.70 – 1.62)	1.02 (0.65 – 1.62)
25-29 (ref)	330 (51.1)	1	1
> = 30	128 (19.8)	1.0 (0.63 – 1.57)	0.92 (0.56 – 1.51)
**Prison – ever**	382 (59.2)	0.94 (0.66 – 1.32)	-
**Length of injecting career (years)**	10.1 (median)	1.00 (0.96 – 1.04)	-
**Injected heroin - past month**	527 (81.7)	0.84 (0.55 – 1.30)	-
**Injected methamphetamines - past month**	203 (31.5)	1.25 (0.87 – 1.79)	-
**Used benzodiazepines - past month**	350 (54.3)	1.25 (0.89 – 1.76)	-
**Injected daily or more**	191 (29.6)	0.56 (0.38 – 0.84)	0.59 (0.38 – 0.91)
**Tested for Hepatitis C / HIV - ever**	594 (92.1)	2.35 (1.08 – 5.11)	-
**Experienced barrier/s to accessing treatment**	72 (11.2)	1.63 (0.98 – 2.70)	1.28 (0.73 – 2.27)
**Currently on OST**	222 (34.4)	1.10 (0.77 – 1.57)	0.94 (0.63 – 1.41)
**Number of other health services used - past month**			
None	422 (65.4)	0.68 (0.46 – 0.99)	0.88 (0.58 – 1.35)
Less than 3 (ref)	178 (27.6)	1	1
3 or more	45 (7.0)	2.30 (1.19 – 4.47)	1.88 (0.93 – 3.80)
**Social/ welfare worker contact - past month**	133 (20.6)	2.43 (1.63 – 3.60)	1.92 (1.24 – 2.98)
**Heroin overdose – 6 months**	63 (9.8)	0.88 (0.49 – 1.58)	-
**Intentional overdose - ever**	73 (11.3)	2.19 (1.34 – 3.60)	1.59 (0.91 – 2.78)
**AUDIT-C score (0 – 11)**			
abstinent (ref)	228 (35.4)	1	11.33 (0.85 – 1.86)
<8	292 (45.3)	1.74 (1.17 – 2.57)	1.10 (0.63 – 1.91)
> = 8	124 (19.2)	1.49 (0.91 - 2.44)	
**SF8 Physical wellbeing score poor (45 or below)**	194 (30.1)	1.47 (1.02 – 2.11)	1.22 (0.80 – 1.86)
**SF8 Mental wellbeing score poor (45 or below)**	317 (49.1)	1.57 (1.12 – 2.22)	1.07 (0.71 – 1.62)
**Personal Wellbeing Index score**	55 (median)	0.99 (0.98 – 1.00)	-
**One or more general health conditions**	336 (52.1)	1.76 (1.24 – 2.49)	1.38 (0.93 – 2.05)

^a^Results are from logistic regression with a final model comprising 645 observations.

^b^AOR reported for exposures significant (p < .1) at the bivariate level which did not exhibit multicollinearity in the final model (overall VIF = 1.78, Pearson chi-square p = .271).

Few associations remained significant in the multivariate analysis. Low socioeconomic status, as indexed by low income, and frequent injecting (characterised by injecting at least daily) were associated with a lower likelihood of GP service utilisation, after adjusting for other variables listed in Table 1. In contrast, living with children and past month contact with a social worker were both associated with an increased likelihood of GP contact. Although having ever been tested for a BBV was significant in the final model, it was removed due to multicollinearity (VIF 7.61). The final model showed a low level of multicollinearity (overall VIF = 1.78).

## Discussion

In this study we have examined GP-related general health service utilisation among PWID. By distinguishing this from primary care attendances exclusively related to drug dependence treatment (including OST), this is the first study to examine factors associated with non-OST related primary care utilisation among a cohort of Australian PWID. Approximately a third of our sample reported accessing a GP for a general health issue in the previous month. While our data are not directly comparable due to the younger age distribution of the study population, it would appear that our sample of Australian PWID frequent GPs more often than the general population [37]. Given that over half of participants reported one or more health conditions, and almost half rated their health and wellbeing poorly, there is clearly a need for non-OST related primary care. This need would be expected to increase over time due to ageing and exacerbation of health issues associated with long term drug use. Examining potential barriers and enablers to service use will contribute towards identifying subpopulations who have limited access to GP-related services.

Our participants with an income below the Australian population average, were considerably less likely to access general health services as compared to those with higher incomes. This finding, consistent with other international research that shows low income is negatively associated with doctor contact [38,39], was evident despite the universal coverage of health services in Australia and the availability of free services for PWID. Countries with similar health systems see more primary care service use among lower socioeconomic groups [40,41], suggesting that there may be systematic differences between PWID and the general population that influence health service utilisation. In this health systems context, low income may be a marker of the impact of other issues such as the geographic access, cost of transport and competing priorities such as obtaining regular meals that we did not measure. The complex vulnerabilities associated with low income, which affects a large proportion of PWID, influences the priority that health takes and consequently the use of health services [42].

Our findings indicate that participants who reported injecting daily or more frequently were almost half as likely to access GPs. More frequent injecting is associated with more severe dependence [43], lower uptake of OST [8,44] and longer time to injecting cessation [43]. Daily injecting has also been associated with multiple and serious injecting-related injuries and diseases [2,45] which are common among PWID in Australia [45]. Despite these risks, the more frequent injectors in our study were less likely to access GP services. While these data suggest that they may be accessing hospital [46] or other services for these complications, we did not observe a significant correlation between frequent injecting and use of emergency or hospital inpatient or outpatient services (data not shown). As such, the healthcare needs of high frequency injectors in our study may remain unmet.

In contrast with the common view of chaotic drug use [47], parenting can represent a point of stability in the lives of PWID [42]. Our study suggests that participants who lived with children were twice as likely to report GP access compared to those who did not. This is consistent with literature around the increased sense of responsibility associated with having children, and the importance PWID place in regular monitoring of their children by health service providers, although this is not always the case [48,49]. However, as the purpose of the visit (if it was for the child or themselves) was not recorded, we were limited in our interpretation of the data beyond access to services. Collaborative services which attend to the needs of parent and child may encourage better uptake of services [50] and increased likelihood that health needs are met. Further research into the impact of having children on health service utilisation and the potential opportunities provided by more frequent health systems contacts for drug using parents and their children is required.

Contact with a social worker in the past month was associated with an increased likelihood of GP service utilisation. PWID access social services for a range of issues such as homelessness and mental health problems [51]. Contact with social services may occur through referral by GPs, conversely, social services may refer PWID to primary care [52,53], meaning that the causal pathway of service access is unclear. Irrespective, the increased likelihood of contact with a GP associated with social worker access indicates that social workers may be an effective gateway to comprehensive health services for an otherwise hard to reach population.

This study is limited firstly by the self-report data used which may be subject to recall and social acceptability bias [54]. Multiple sampling methods may limit representativeness of this cohort, and hence the generalizability of these findings. In addition, the distinction between GPs situated at general and targeted services may have been subject to some misclassification as visits to PWID-specific primary care centres were included on the basis of descriptive text and each visit could include a range of activities, meaning that some GP-related visits may have been missed. However this is also an advantage as descriptive text contextualises the reason for the service use and has been provided by the recipient of the service. Future studies of PWID primary care need to clarify the purposes of all GP visits. We are limited in interpreting the causal pathway between the exposures and outcomes due to the cross-sectional nature of this study, for example if physical and mental wellbeing would increase access to GP-related sevices, or vice versa. We plan to address these gaps using longitudinal analysis.

## Conclusions

Building on broader research around health service utilisation among PWID, our study focuses on general health services as a principal element in determining health outcomes for this population. The strength of this research lies in the sample itself who are a relatively young cohort of actively injecting PWID, the majority of whom were out of treatment at baseline and may have different health needs and service utilisation patterns compared to other injecting populations previously studied e.g. [7,55]. Given the high rates of GP service utilisation, the health needs of this population are not limited to opioid dependence, and consequently health service needs extend beyond OST. Ensuring PWID have access to health services early in their injecting career could prevent chronic conditions prevalent among their older counterparts [56]. It may also reduce the use of emergency services for health needs that could be addressed in primary care settings [57]. Key findings from this study are that low socioeconomic status and frequent injecting practices impair GP service utilisation within this population. The former is particularly notable; providing healthcare cards to PWID of low socioeconomic status may not be sufficient to support access to services. Comprehensive services and support can alleviate the pressures faced by this population and create an environment conducive to harm minimisation strategies. Further research using longitudinal data from this cohort will serve to understand health service utilisation trends and supplement our current findings.

## Competing interests

The authors declare that they have no competing interests.

## Authors’ contributions

DN and PD developed the analysis plan. DN conducted the data analysis, and led the writing of the manuscript. PD and MS contributed to the interpretation of the data, and approved the final manuscript. All authors read and approved the final manuscript.

## Pre-publication history

The pre-publication history for this paper can be accessed here:

http://www.biomedcentral.com/1472-6963/14/308/prepub
